# Lumbar and pelvis movement comparison between cross-court and long-line topspin forehand in table tennis: based on musculoskeletal model

**DOI:** 10.3389/fbioe.2023.1185177

**Published:** 2023-06-19

**Authors:** Yuqi He, Minjun Liang, Yufei Fang, Gusztáv Fekete, Julien S. Baker, Yaodong Gu

**Affiliations:** ^1^ Research Academy of Medicine Combining Sports, Ningbo No.2 Hospital, Ningbo, China; ^2^ Faculty of Engineering, University of Pannonia, Veszprém, Hungary; ^3^ Savaria Institute of Technology, Eötvös Loránd University, Szombathely, Hungary; ^4^ Faculty of Sports Science, Ningbo University, Ningbo, China; ^5^ Department of Sport, Physical Education and Health, Hong Kong Baptist University, Hong Kong, China

**Keywords:** pelvis rotation, lumbar movement, opensim, musculoskeletal model, trunk rotation, topspin forehand, table tennis

## Abstract

**Introduction:** Cross-court and the long-line topspin forehand is the common and basic stroke skill in table tennis. The purpose of this study was to investigate the differences in lumbar and pelvis movements between cross-court and long-line topspin forehand strokes in table tennis based on musculoskeletal demands using OpenSim.

**Materials and Methods:** The eight-camera Vicon system and Kistler force platform were used to measure kinematics and kinetics in the lumbar and pelvis movement of sixteen participants (Weight: 69.89 ± 1.58 kg; Height: 1.73 ± 0.03 m; Age: 22.89 ± 2.03 years; BMI: 23.45 ± 0.69 kg/m^2^; Experience: 8.33 ± 0.71 years) during cross-court and long-line topspin forehand play. The data was imputed into OpenSim providing the establishment of the Giat2392 musculoskeletal model for simulation. One-dimensional statistical parametric mapping and independent samples t-test was performed in MATLAB and SPSS to analyze the kinematics and kinetics.

**Results:** The results show that the range of motion, peak moment, and maximum angle of the lumbar and pelvis movement in cross-court play were significantly higher than in the long-line stroke play. The moment of long-line in the sagittal and frontal plane was significantly higher than cross-court play in the early stroke phase.

**Conclusion:** The lumbar and pelvis embody greater weight transfer and greater energy production mechanisms when players performed cross-court compared to long-line topspin forehand. Beginners could enhance their motor control strategies in forehand topspin skills and master this skill more easily based on the results of this study.

## Introduction

Topspin forehand is regarded as one of the most attacking strokes in table tennis based on the high speed and fast rotation (Poizat et al., 2004; Qian et al., 2016; He et al., 2020; He et al., 2022a). The mastery of the topspin forehand is also considered an important factor in differentiating elite athletes (Iino and Kojima, 2011; He et al., 2020). The biomechanical mechanisms inherent in topspin forehand have been extensively reported in previous studies, such as the joint kinematics (Iino and Kojima, 2009; He et al., 2020; He et al., 2021a) and kinetics (Iino and Kojima, 2011), ground reaction force (GRF) (Zhou et al., 2021a), plantar pressure (Fu et al., 2016; Lam et al., 2019; He et al., 2021b), and electromyography (EMG) and information regarding muscle contraction dynamics (Le Mansec et al., 2018; Chen et al., 2022). These studies amply illustrate the value and significance of investigating the biomechanical intrinsic mechanisms of the topspin forehand.

The speed and accuracy of the ball, as well as the success rate, are the key determinants of the quality of strokes (Landlinger et al., 2012; Kolman et al., 2019). The key role of ball speed in table tennis supports the optimization of stroke skills and distinguishes the performance level of players. Trunk rotation was strongly correlated with the velocity of the racket (Akutagawa and Kojima, 2005). Due to the critical role of pelvic movement on the axial rotation of the trunk rotation and hitting speed, pelvic movement plays a crucial role in powerful hitting sports, such as table tennis, tennis, and baseball (Elliott and Zatsiorsky, 2000; Akutagawa and Kojima, 2005). In table tennis, the horizontal velocity of the racket during the topspin forehand stroke was benefited by the peak angular velocity of pelvic axial rotation and the pelvic axial rotation torque on the playing hand side (Iino, 2018; He et al., 2022a). In order to remain powerful and competitive, players need to increase the acceleration of their playing hand by optimizing their stroke skill and the efficiency of the power chain transmission, which brings gains to the spin effect and flight speed of the ball during the topspin forehand (Qian et al., 2016; Lam et al., 2019; He et al., 2020; Chen et al., 2022). In tennis, the rotational movement of the lower trunk in the horizontal plane was very frequent (Muyor et al., 2013). In baseball, the rotation of the trunk and leg has been reported as the important power source in ground strokes (Elliott and Zatsiorsky, 2000; Akutagawa and Kojima, 2005). Even though a great deal of previous research and evidence has strongly supported that the development of the optimal trunk rotation was one of the most important factors to master the topspin strokes (Akutagawa and Kojima, 2005), however, the movement characteristics of the human pelvis have hardly been specifically studied and reported in table tennis topspin forehand stroke.

The development of musculoskeletal models to investigate the mechanical performance of human structures during movement is an important and commonly used tool in the field of biomechanics research. Motion reconstruction based on captured data allows access to additional variables of interest to explain some phenomena and intrinsic patterns. Examples include the reconstruction of musculoskeletal models in OpenSim and Visual3D to calculate joint kinematics, kinetics, and muscle forces during movement (Seth et al., 2018; Dorschky et al., 2019; Nitschke et al., 2020). Iino (2018) created a musculoskeletal model in OpenSim and investigated the muscular effort of lower limb muscles during the topspin forehand stroke (Iino et al., 2018). Yang et al. (2022) calculated the angles and moments of lower limb joints of 36 elite table tennis players during chasse-step footwork by OpenSim to investigate the gender difference (Yang et al., 2022). He et al. (2022a) investigated lower limbs muscle force, joint kinematics, and joint kinetics in footwork during the topspin forehand using OpenSim (He et al., 2022b). That evidence suggests simulation using a musculoskeletal model to obtain key information is viable and recognized for explaining the mechanisms underlying table tennis topspin forehand stroke movements.

As a typical one-handed racket sport, table tennis has been proven to have a bad effect on the symmetry of the trunk, and the imbalance of symmetry is one of the key factors leading to sports injuries (Bańkosz and Barczyk-Pawelec, 2020). The revealing of the inner mechanism of the lumbar and pelvis movement during stroke play in table tennis can provide reference information for exploring sports injuries caused by symmetry imbalance. The constant interplay of technical and tactical skills is crucial to winning each point in a competition game (Kolman et al., 2019). To achieve tactical goals, athletes need to perform specific skills (Kolman et al., 2019). The importance of the cross-court (CC) topspin forehand is reflected in the fact that the CC has always been the object or vehicle of study in previous studies on the biomechanics of table tennis (Iino and Kojima, 2011; Malagoli Lanzoni et al., 2018; He et al., 2020; He et al., 2021a; Xing et al., 2022). Besides, the long-line (LL) topspin forehand, as one of the basic strokes in racket sports, has been widely studied not only in table tennis but also in tennis (Landlinger et al., 2010; Pedro et al., 2022). The functional role of the topspin forehand stroke skills in influencing tactics results in players being able to optimize their skills to enhance the success of their tactics and further ensure an advantageous position in the match. Although previous studies have investigated the kinematics difference between CC and LL (Malagoli Lanzoni et al., 2018; He et al., 2020), the lumbar and pelvis movement and the kinetic information have not been measured and analyzed. To summarize, this study aimed to simulate a musculoskeletal model using OpenSim software to investigate the difference in lumbar and pelvis movements between CC and LL topspin forehand strokes in table tennis. Firstly, this study can be applied to guide the coaching and training of table tennis players, especially for beginner players to recognize the role of lumbar and pelvis movement in optimizing topspin forehand stroke skills for application in the game. Secondly, the movement information could be provided to explore the injury risk in table tennis or other racket sports. The hypothesis of this study was that CC and LL topspin forehand show significantly different kinematics and kinetics, and the difference would be evident in the transverse plane.

## Methods

### Participants

Sixteen male table tennis players from the Ningbo University table tennis team volunteered to participate in this study and provided written informed consent after the purpose and process of this study were explained. All the participants were at the national-one performance level and right-handed, as well as free from any neuromuscular injury in the past 6 months. The demographic information of participants is shown in Table 1. The Ethics Committee of Ningbo University approved this study.

**TABLE 1:** Demographic information.

Weight (Kg)	Height (M)	Age (Y)	BMI (Kg/m^2^)	Experience (Y)
69.89 ± 1.58	1.73 ± 0.03	22.89 ± 2.03	23.45 ± 0.69	8.33 ± 0.71

### Experimental protocol and equipment

**FIGURE 1:**
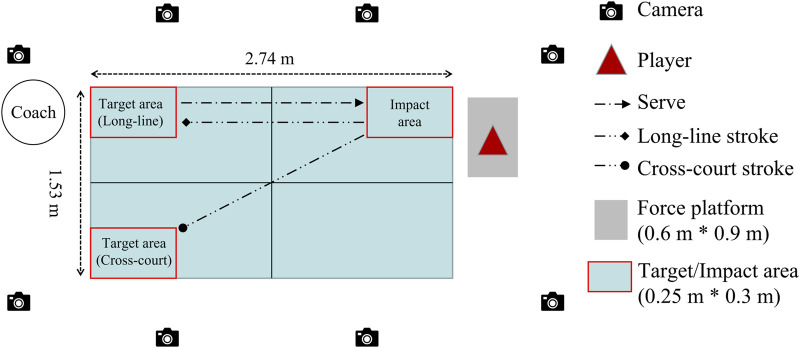
Experimental environment and set-up.

The experiment was performed in the biomechanics laboratory of the Ningbo University Research Academy of Grand Health. As shown in Figure 1, the kinematics of participants were captured by an eight-camera Vicon motion capture system (Oxford Metrics, Ltd., Oxford, United Kingdom) which was set at the sampling frequency of 200 Hz. The kinetics of participants was recorded by a force platform (Kistler, Switzerland) using a sampling frequency of 1,000 Hz. All devices used for data acquisition were electronically connected to achieve the multi-parameter synchronous acquisition of the test data. The Gait2392 model was selected to simulate the movement of the participant in the OpenSim (Stanford University, Stanford, CA, United States), and the thirty-nine reflective markers (12.5 mm in diameter) placement was replicated according to the previous studies (Delp et al., 2007).

Participants used uniform rackets (Butterfly Tenergy 05 Max and DHC Hurricane 3 rubber sheets), balls (D40+, Double Happiness Sports Company, Shanghai, China), and playing table (Rainbow, Double Happiness Sports Company, Shanghai, China), as well as match table tennis shoes and tights during the experiment.

### Procedure

Prior to the commencement of the formal test, participants were allowed to complete 5 min of static stretching and 10 min of running in a spacious area to warm up. Subjects were required to stand on the force platform to complete the static coordinates collection process after putting the reflective markers on the subjects’ bodies. To check the operation of all the equipment and help the subjects quickly familiarize themselves with the laboratory environment, subjects were asked to perform five topspin forehand stroke tasks before the formal data collection session.

As shown in Figure 1, in the formal test, the coach was shooting the ball with normal service to the impact area (0.25 m * 0.3 m). The subject stands on the right side of the playing table and was required to perform the topspin forehand stroke to return the ball to the long-line target area (0.25 m * 0.3 m) and cross-court target area (0.25 m * 0.3 m), respectively. The CC topspin forehand started first, then perform the LL topspin forehand. There is no rest time during the formal test until the successfully recorded 5 trials data of the CC and the LL topspin forehand for each participant, respectively. The subject and a qualified coach judged the quality of motion during the test. The test data were excluded if the drop point of the ball was out of the target area and the motion quality was questioned. Meanwhile, the data performance was also used to evaluate the validity of data collection. The size set of the impact and target area was as same as in previous studies (Zhou et al., 2021a; He et al., 2022b).

### Definition

In this study, only the data in the forward swing phase during the stroke were collected and analyzed. The pelvis movement in the transverse plane was defined as pelvis axial rotation (PAR), as well as the lumbar movement in the sagittal, frontal, and transverse plane, was defined as lumber flexion (LF), lumbar left lateral bending (LLB), and lumbar axial rotation (LAR) in this study.

**FIGURE 2:**
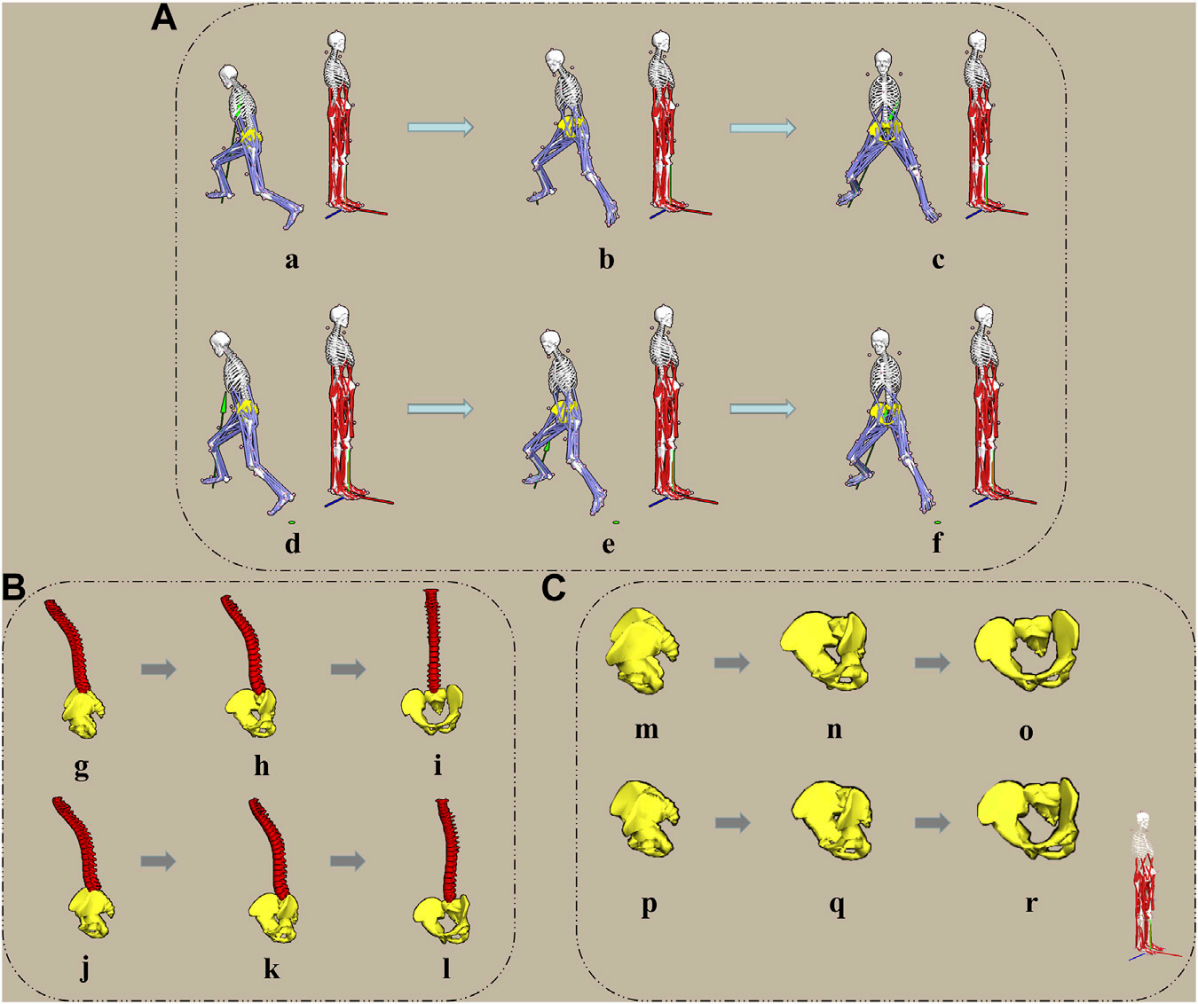
Diagram of the human musculoskeletal model of the CC and LL topspin forehand stroke. **(A)** indicate the topspin forehand stroke process. (a–c) and (d–f) indicate the CC and LL, respectively. **(B)** shows the lumbar and pelvis movement during the topspin forehand stroke. (g–i) and (j–l) indicate the CC and LL, respectively. **(C)** shows the pelvis movement during the topspin forehand stroke. (m–o) and **(**p–r**)** indicate the CC and LL, respectively.

As shown in Figure 2, “A”, “B”, and “C” are the CC and LL topspin forehand stroke process in the full body, lumbar, and pelvis view, respectively. Besides, the “a-c”, “g-I”, and “m-o” in CC and “d-f”, “j-l”, and “p-r” in LL indicate the “end of the backward swing (EB)”, “medium forward swing (MF)”, and “end of the forward swing (EF)”, respectively. The definition of EB, MF, and EF was completed in the Vicon Nexus 1.8.6 software (Oxford Metrics, Ltd., Oxford, United Kingdom). When the GRF wave reached the first peak value was defined as EB. After the first peak in the GRF wave, the medium value was defined as MF. And the second peak value in the GRF wave was defined as EF.

### Data processing

**FIGURE 3:**
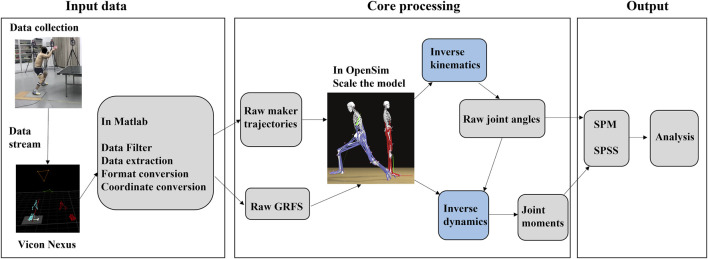
Flow chart and data processing.

As shown in Figure 3, GRF and kinematic data during CC and LL topspin forehand were identified and acquired using Vicon Nexus 1.8.6 software. The data was exported from the Vicon Nexus with a c3d. format file, and use MATLAB R2019a (The MathWorks, Natick, MA, United States) to perform coordinate system conversion, lower pass filtering, data extraction, and format conversation for all data. The detailed process in MATLAB R2019 has been outlined in previous studies (Zhou et al., 2021b; He et al., 2022b) that as follows: convert the coordinate to the subsequent simulation coordinate system, filter the marker trajectory and the GRF, and convert the formats of data to the trc. and mot. formats that are required by OpenSim. The statics model of the subjects was imported into OpenSim and the anthropometric model was obtained. Then we identified the muscle’s starting and ending points and ensured the moment arms were consistent with the length of the subject’s limb (Delp et al., 2007; He et al., 2022b). We used the inverse kinematic tool (IK) to calculate the kinematics data of the subject during CC and LL topspin forehand and created a motion file using mot format. We then imported the GRF and markers files using the inverse dynamics tool (ID) and calculated the joint moment. In OpenSim, the weighted least square problem was solved by the IK function to minimize the distance of markers’ placements between the experimental and virtual; the generalized positions, velocities, and accelerations defined the motion of the model, which resulted in the unknown generalized forces were calculated by those known motion variables.

### Statistical analysis

Kinematics and the moment of the pelvis and lumbar were analyzed by one-dimensional statistical parametric mapping (SPM1d) analysis in MATLAB R2019a. The Rom and peak moment of the pelvis and lumbar were analyzed by independent samples t-test in SPSS 24.0 (SPSSs Inc., Chicago, IL, United States). In the SPM analysis, we performed the custom script in MATLAB to expend all data into a time series curve of 101 data points. The significance level in this study was set as *p* < 0.05.

## Result

### Lumbar movement

**FIGURE 4:**
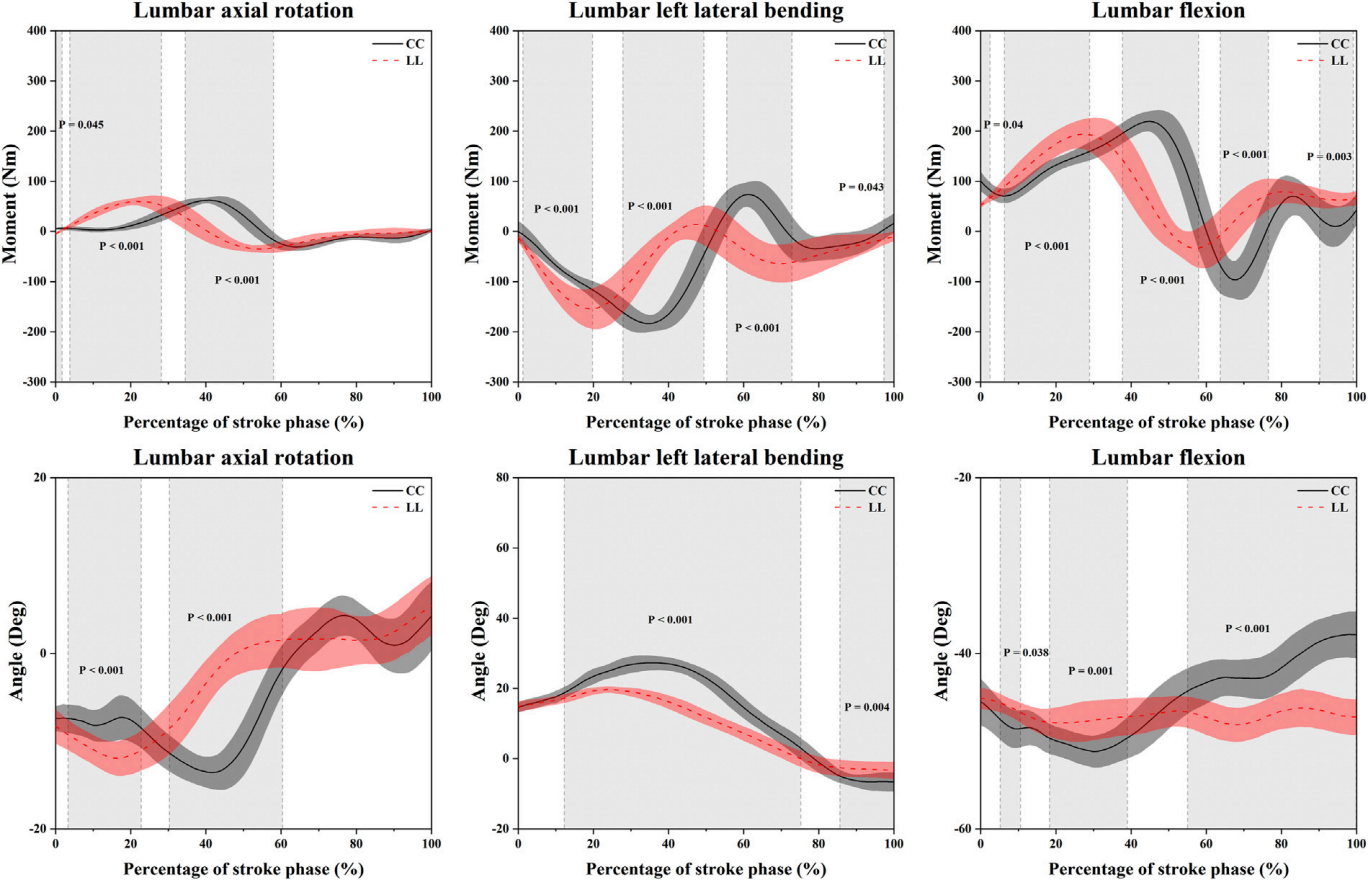
Illustration of the result of the angle and moment in the LAR, LLB, and LF between the CC and LL topspin forehand showing the SPM1d outputs. Grey-shaded areas indicate that there are significant differences (*p* < 0.05) between the CC and LL. LL indicates long-line topspin forehand, CC indicates cross-court topspin forehand.

Table 2; Figure 4 show the SPM1d analysis result of the angle and moment in the LAR, LLB, and LF between the CC and LL topspin forehand. In the LAR, the LL showed a significantly higher moment than CC in the 0%–1.75% (*p* = 0.045, *t* = 3.331) and 3.80%–28.14% (*p* < 0.001, *t* = 3.331) phase, and a significantly higher angle in 3.30%–22.79% (*p* < 0.001, *t* = 3.129) phase. However, the LL showed a significantly lower moment and angle in the 34.51%–57.98% (*p* < 0.001, *t* = 3.331) and 30.29%–60.43% (*p* < 0.001, *t* = 3.129) phase than CC, respectively. In the LLB, CC showed a significantly higher moment than LL in the 27.93%–49.48% (*p* < 0.001, *t* = 3.258), 55.56%–72.87% (*p* < 0.001, *t* = 3.258), 97.38%–100% (*p* = 0.043, *t* = 3.258) phase, and a significantly higher angle in the 12.29%–75.30% (*p* < 0.001, *t* = 3.125) and 85.68%–100% (*p* = 0.004, *t* = 3.125) phase. The LL showed a significantly higher moment in the 1.30%–19.81% (*p* < 0.001, *t* = 3.258) phase than CC. In the LF, the moment of LL was significantly higher than CC in the 6.38%–29.08% (*p* < 0.001, *t* = 3.344) and 90.29%–99.25% (*p* = 0.003, *t* = 3.344) phase, and the angle were higher than CC in the 55.13%–100% (*p* < 0.001, *t* = 3.08) phase. The CC showed a significantly higher moment in the 0%–2.52% (*p* = 0.04, *t* = 3.344), 37.81%–58.06% (*p* < 0.001, *t* = 3.344), and 63.86%–76.60% (*p* < 0.001, *t* = 3.344) phase, and a significantly higher angle in the 5.26%–10.63% (*p* = 0.038, *t* = 3.080) and 18.40%–39.10% (*p* = 0.001, *t* = 3.080) phase than LL.

**TABLE 2:** The moment and angle results of the SPM analysis. (Unit: %).

Variables	Percentage (*p*)
LAR Moment	0–1.75 (0.045), 3.80–28.14 (<0.001), 34.51–57.98 (<0.001)
LLB Moment	1.30–19.81 (<0.001), 27.93–49.48 (<0.001), 55.56–72.87 (<0.001), 97.38–100 (0.043)
LF Moment	0–2.52 (0.04), 6.38–29.08 (<0.001), 37.81–58.06 (<0.001), 63.86–76.6 (<0.001), 90.29–99.25 (0.003)
PAR Moment	4.15–30.01 (<0.001), 45.01–80.63 (<0.001),
LAR Angle	3.30–22.79 (<0.001), 30.29–60.43 (<0.001)
LLB Angle	12.29–75.30 (<0.001), 85.68–100 (0.004),
LF Angle	5.26–10.63 (0.038), 18.40–39.10 (0.001), 55.13–100 (<0.001)
PAR Angle	0–1.69 (0.049), 10.29–78.31 (<0.001), 88.90–100 (0.027)

Note: the percentage indicates the process of the stroke play phase.

**FIGURE 5:**
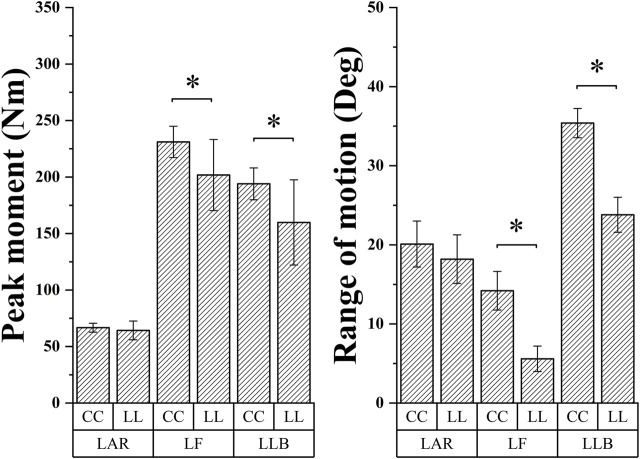
The Rom and peak moment comparison of lumbar movement between the CC and LL topspin forehand. “*” indicate a significant difference between LL and CC. LL indicates long-line topspin forehand, CC indicates cross-court topspin forehand. LAR indicates lumbar axial rotation, LLB indicates lumbar left lateral bending, and LF indicates lumbar flexion.

As shown in Table 3; Figure 5, the Rom and peak moment of LLB and LF in CC were significantly higher than LL (Rom: *t* = 16.55, *p* = 0; *t* = 12.139, *p* = 0. Peak moment: *t* = −3.396, *p* = 0.002; *t* = 3.412, *p* = 0.003). The maximum LAR, LLB, and LF in the CC were significantly higher than LL (*t* = −2.84, *p* = 0.008; *t* = 13.206, *p* = 0; *t* = −3.307, *p* = 0.003).

**TABLE 3:** Comparison of Rom, Peak moment, and Maximum angle of lumbar and pelvis movement between CC and LL topspin forehand.

		Rom (Deg)				Peak moment (Nm)				Maximum angle (Deg)			
		Mean ± SD	*t*	*p*	*95%CI*	Mean ± SD	*t*	*p*	*95%CI*	Mean ± SD	*t*	*p*	*95%CI*
LAR	CC	20.10 ± 2.91	1.853	0.073	−1.90, 4.00	66.69 ± 3.97	1.06	0.30	−2.26, 7.13	−14.13 ± 1.80	−2.84	0.008*	−2.83, −0.47
	LL	18.19 ± 3.07				64.25 ± 8.29				−12.48 ± 1.57			
LLB	CC	35.39 ± 1.85	16.55	0*	10.16, 13.02	−194.0 ± 14.11	−3.396	0.002*	−54.72, 13.62	27.78 ± 2.19	13.206	0*	6.55, 8.94
	LL	23.80 ± 2.22				−159.82 ± 37.70				20.04 ± 1.03			
LF	CC	14.20 ± 2.44	12.139	0*	7.16, 10.06	231.10 ± 13.87	3.412	0.003*	11.43, 47.19	−51.46 ± 1.77	−3.307	0.003*	−2.84, −0.67
	LL	5.59 ± 1.61				201.79 ± 31.44				−49.71 ± 1.28			
PAR	CC	75.96 ± 5.64	12.798	0*	19.08, 26.31	597.68 ± 115.84	2.245	0.034*	6.01, 143.78	−52.02 ± 3.31	−9.627	0*	−15.08, −9.81
	LL	53.26 ± 4.65				522.78 ± 66.27				−39.57 ± 4.17			

Note: “*” indicate a significant difference between LL and CC. LL indicates long-line topspin forehand, CC indicates cross-court topspin forehand. LAR indicates lumbar axial rotation, LLB indicates lumbar left lateral bending, LF indicates lumbar flexion, and PAR indicates pelvis axial rotation.

### Pelvis axial rotation

**FIGURE 6:**
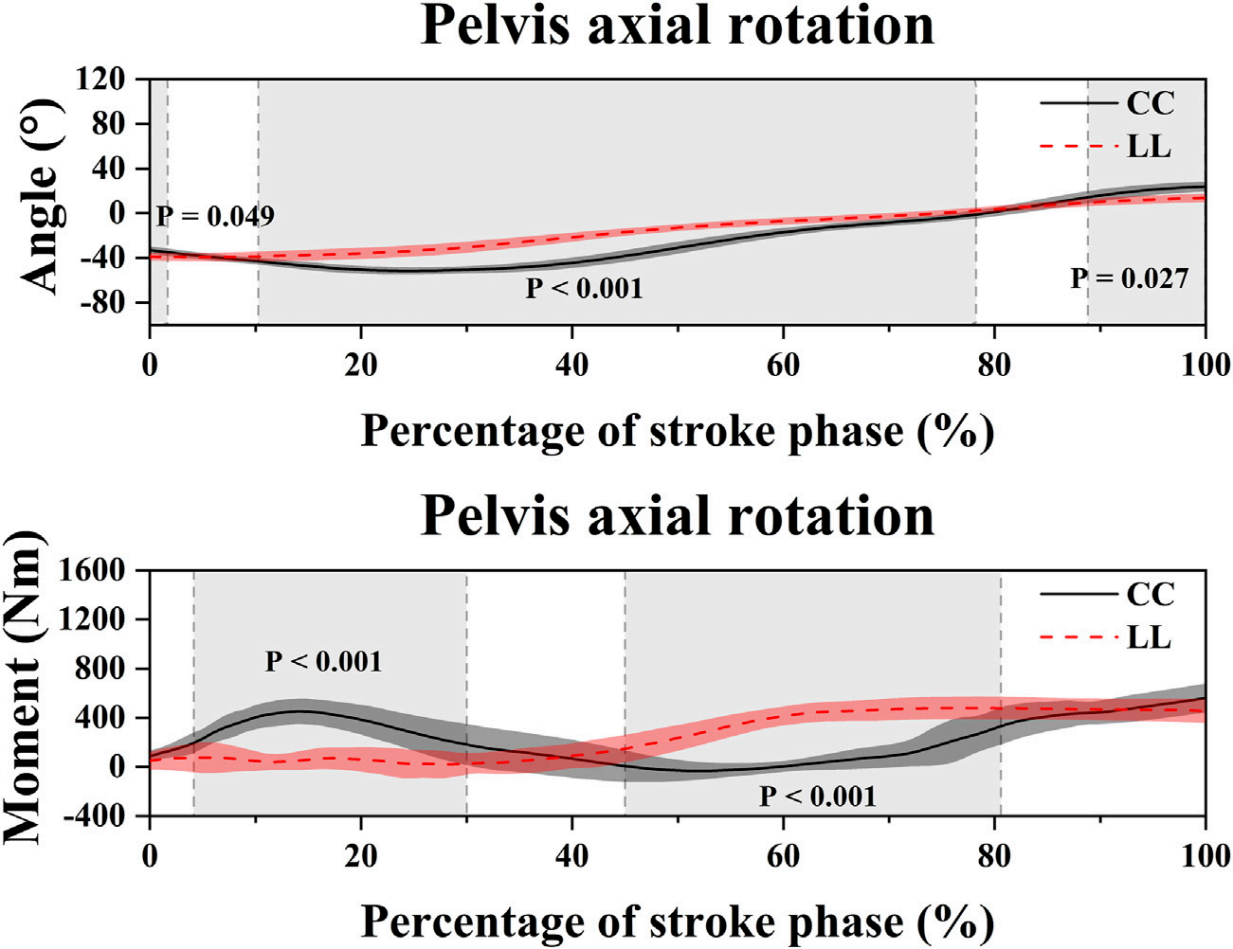
Illustration of the result of angle and moment of the PAR between the CC and LL topspin forehand showing the SPM1d outputs. Grey-shaded areas indicate that there are significant differences (*p* < 0.05) between the CC and LL. LL indicates long-line topspin forehand, CC indicates cross-court topspin forehand.

Table 2; Figure 6 show the SPM1d analysis result of the angle and moment of PAR between the CC and LL topspin forehand. The PAR angle of CC was significantly higher than LL in the 10.29%–78.31% (*p* < 0.001, *t* = 2.86) and 88.90%–100% (*p* = 0.027, *t* = 2.86) phase, but significantly lower than LL in 0%–1.69% (*p* = 0.049, *t* = 2.86) phase. The PAR moment of CC was significantly higher than LL in the 4.15%–30.01% (*p* < 0.001, *t* = 3.288) phase and significantly lower than LL in the 45.01%–80.63% (*p* < 0.001, *t* = 3.288) phase.

**FIGURE 7:**
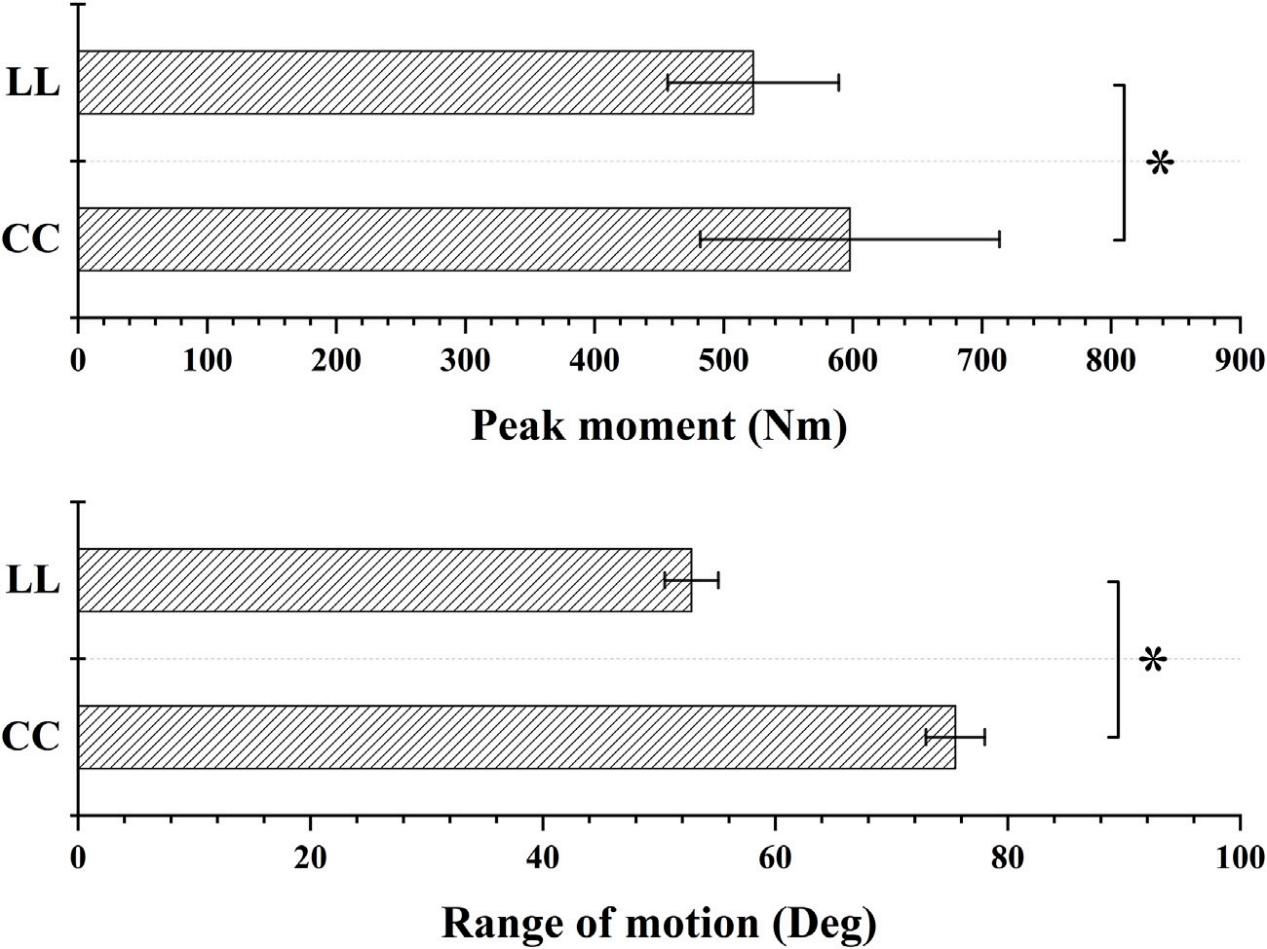
The Rom and peak moment comparison of PAR between CC and LL topspin forehand. “*” indicate a significant difference between LL and CC. LL indicates long-line topspin forehand, CC indicates cross-court topspin forehand.

As shown in Table 3; Figure 7, The maximum PAR in the CC was significantly higher than LL (*t* = −9.627, *p* = 0), and Rom and peak moment of PAR in the CC was significantly higher than LL (*p* = 0, *t* = 12.798; *p* = 0.034, *t* = 2.245).

## Discussion

This study simulated the musculoskeletal model in OpenSim to investigate the lumbar and pelvis movement difference between the CC and LL topspin forehand in table tennis. The key finding of this study was (Qian et al., 2016) the main difference between CC and LL topspin forehand in the lumbar movement was found in the LLB and LF, the Rom, peak moment, and maximum angle of the LLB and LF in CC were significantly higher than LL (He et al., 2022a); the Rom, peak moment and maximum angle of PAR in CC were significantly higher than LL (Poizat et al., 2004); the moment of LL in the LF and LLB was significantly higher than CC in the early stroke phase. The results of the current study were consistent with our hypothesis, the CC and LL showed a significant difference in lumbar and pelvis movement in the transverse plane. Investigating the difference in lumbar and pelvis movement between the CC and LL topspin forehand could provide guidelines for coaches and players to understand the mechanisms inherent from a biomechanical perspective, especially the information could help beginners to build awareness of CC and LL topspin forehand skills more easily for enhance their stroke skill and motor control.

The lumbar movement is widely focused, especially in racket sports. The Rom and maximum angle of the LLB and LF in CC were significantly higher than LL in this study. This could be explained by the fact that the target area in CC is the left side of the playing body, and the players need to adjust their bodies to hit the ball correctly. A higher LLB Rom and maximum angle could bring a completed body weight transfer which could benefit the energy transfer from the trunk to the upper limb following the proximal-to-distal segmental sequences in the kinetic chain (Fu et al., 2016; Bańkosz and Winiarski, 2018; Lam et al., 2019; He et al., 2022b). A higher LF in CC probably means a more forward shift of the center of gravity in the sagittal plane, furthermore, the shift in the center of gravity will result in greater energy transfer, which may mean greater racket acceleration during the forward swing phase. In previous studies, lower back pain (LBP) in athletes of racket sports has been thought to be closely associated with lumbar movement (Kawasaki et al., 2005; Campbell et al., 2013; Campbell et al., 2014; Connolly et al., 2020; Connolly et al., 2021). The lumbar section as the main core region of the body plays a coordinating role in the compound movement of the upper and lower extremities, however, this is also a major cause of LBP, because in the topspin forehand motor, the LLB, LAR, and LF have occurred simultaneously, the ‘coupled movements’ could bring more pressure and load to vertebral structures than the single plane movement (Gunzburg et al., 1992; Haberl et al., 2004). Previous studies have shown that 32% of athletes experience pain in the lumbar and spinal column during competition or immediately after training, and 36% of athletes even quit training due to pain (He et al., 2022a). In the topspin forehand, the athlete’s unilateral upper extremity needs to hit the ball with maximum force, and this often leads to full body involvement, increasing the impact of the stroke through a large transfer of full body weight. However, the foot on the non-playing side needs to be locked on the ground to maintain dynamic body balance. Extensive repetition of this compensatory movement leads to severe overload of the posterior side of the disc and causes injury. Further, the significantly greater maximum angle and peak moment of LF and LLB exhibited in CC relative to LL may imply a greater risk of injury.

Extensive research on topspin forehand already exists, but few studies have reported detailed information on pelvis movement during topspin forehand stroke. The result shows that the Rom, peak moment and maximum angle of PAR in CC were significantly higher than in LL. The ROM value of PAR in this study was basically consistent with the study of Bańkosz and Winiarski (2018) and Malagoli Lanzoni et al. (2018) respectively, this indicates that during the topspin forehand, the players follow a steady motor program and execute it repeatedly, which may be gradually fixed and standardized in daily training and practice. Players will make small adjustments to their own movement patterns according to the changes in the situation during the match, and finally complete the stroke task. Previous studies have reported the important role of pelvic axial rotation on racket acceleration (Iino, 2018; Xia et al., 2020), even trunk rotation is probably the most critical factor in the development of racket speed (Landlinger et al., 2010), a higher velocity was observed in CC as compared with LL in tennis (Landlinger et al., 2010). The CC has a longer trajectory than the LL (Malagoli Lanzoni et al., 2018), and the target area was on the left side of the playing body, these were the results obtained in players trying to get a racket acceleration during the forward swing phase through full muscle elongation and a greater axial rotation of the lower trunk in CC. The ROM and peak moment of PAR in CC was significantly higher than in LL in the current study, this also could be linked to a more weight transfer that could bring more energy transfer to further enhance the racket acceleration (He et al., 2022b), because the playing arm was the endpoint of the body during stroke motor program which follows the proximal-to-distal segmental sequences in the kinetic chain (Fu et al., 2016; Bańkosz and Winiarski, 2018; Lam et al., 2019; He et al., 2022b). However, the result of the pelvis movement between CC and LL was different from the study of Malagoli Lanzoni et al. (2018). This is due to the different calculations, in their study the angle of axial pelvic movement was calculated relative to the table and not based on the player’s own body, and the position of the player’s feet when hitting the ball was not taken into account, the player’s position was different in CC and LL, so the movement information of the pelvis is not comprehensive enough if only the playing table was used as a reference in evaluation. The moment of LL in the LF and LLB was significantly higher than CC in the early stroke phase. This result could support the hypothesis of Xing et al. (2022) in the discussion section. Furthermore, this could probably be explained that LL has a shorter trajectory (Malagoli Lanzoni et al., 2018) and less forward swing time compared with CC (Xing et al., 2022), which results in the players having to pull their muscles as soon as possible in a limited time to gain more elastic energy to complete an attractive stroke. On the other hand, a shorter running trajectory of the ball in LL means a shorter reaction time for the player, which further requires the player to return to the ready position for the next stroke. This could explain the ROM and maximum angle of LF and LLB in the LL were significantly less than in the CC.

After understanding the differences between the lumbar and pelvis movements of CC and LL topspin forehand, players could enhance the motor control of lumbar and pelvis movements according to the movement characteristics, either by enhancing core strength to improve the explosive power of lumbar and pelvis movements or by flexibility training to enhance lumbar and pelvis synergy, as these modalities are able to enhance the level of energy transfer in the power chain and improve performance. Beginners could quickly understand the role and contribution of the lumbar and pelvis in topspin forehand skills based on the results of this study, thus making it easier to master CC and LL topspin forehand skills.

There are several limitations of this study that have to be mentioned (Qian et al., 2016): the result of this study was limited to male table tennis players; therefore, the result may not be generalizable to female players (He et al., 2022a); the results of this study were generated in a laboratory environment and the results may be inaccurate in relation to a real game environment, for example, where the player needs to judge the rotation and direction of the next ball, which may result in the player having to adjust their body to ensure they can move to the correct position at all time (Poizat et al., 2004); the motion time of stroke in each phase and racket velocity should be measured in further studies.

## Conclusion

This study analyzed and compared the movement of the lumbar and pelvis during the CC and LL topspin forehand. The results showed that the lumbar and pelvis embody greater weight transfer and greater energy production mechanisms when players performed CC compared to LL, while it is important to note that players are also at greater risk of lumbar injury in CC. Beginners could enhance their motor control strategies in forehand topspin skills and master this skill more easily based on the findings of this study.

## Data Availability

The raw data supporting the conclusion of this article will be made available by the authors, without undue reservation.
